# Pubic and ischial sarcoidosis: a single bone lesion

**DOI:** 10.1259/bjrcr.20210091

**Published:** 2022-01-10

**Authors:** Kaoutar Imrani, Kaoutar Znati, Ittimade Nassar, Nabil Moatassim Billah

**Affiliations:** 1 Radiology Department, Ibn Sina University Hospital, Mohammed V University, Rabat, Morocco; 2 Department of anatomo-pathology, Ibn Sina University Hospital, Mohammed V University, Rabat, Morocco

## Abstract

Bone sarcoidosis is very rarely indicative of the disease. When bone lesion is associated with lung and lymph node involvement, diagnosis can be made based on clinical and imaging features. When bone lesion is isolated, it is difficult to differentiate it from bone metastases because they both have similar appearance in imaging: in this case, the diagnosis is made by bone biopsy with histological study. We report the case of a 61-year-old male with a lytic lesion of the right ischio pubic ramus which appears to be aggressive whose biopsy revealed bone sarcoidosis.

## Case report

A 61-year-old patient, with no previous history, presented at the emergency department for lateral right-sided buttock pain that had been evolving for 4 months, inflammatory, calmed by the use of non-steroidal anti-inflammatory drugs. Physical examination was without particularity.

Biological data showed inflammatory syndrome (C-reactive protein = 40 mg l^−1^; erythrocyte sedimentation rate = 45 mm/h. The blood cell counts and the serum protein electrophoresis were normal.

**Figure 1.**
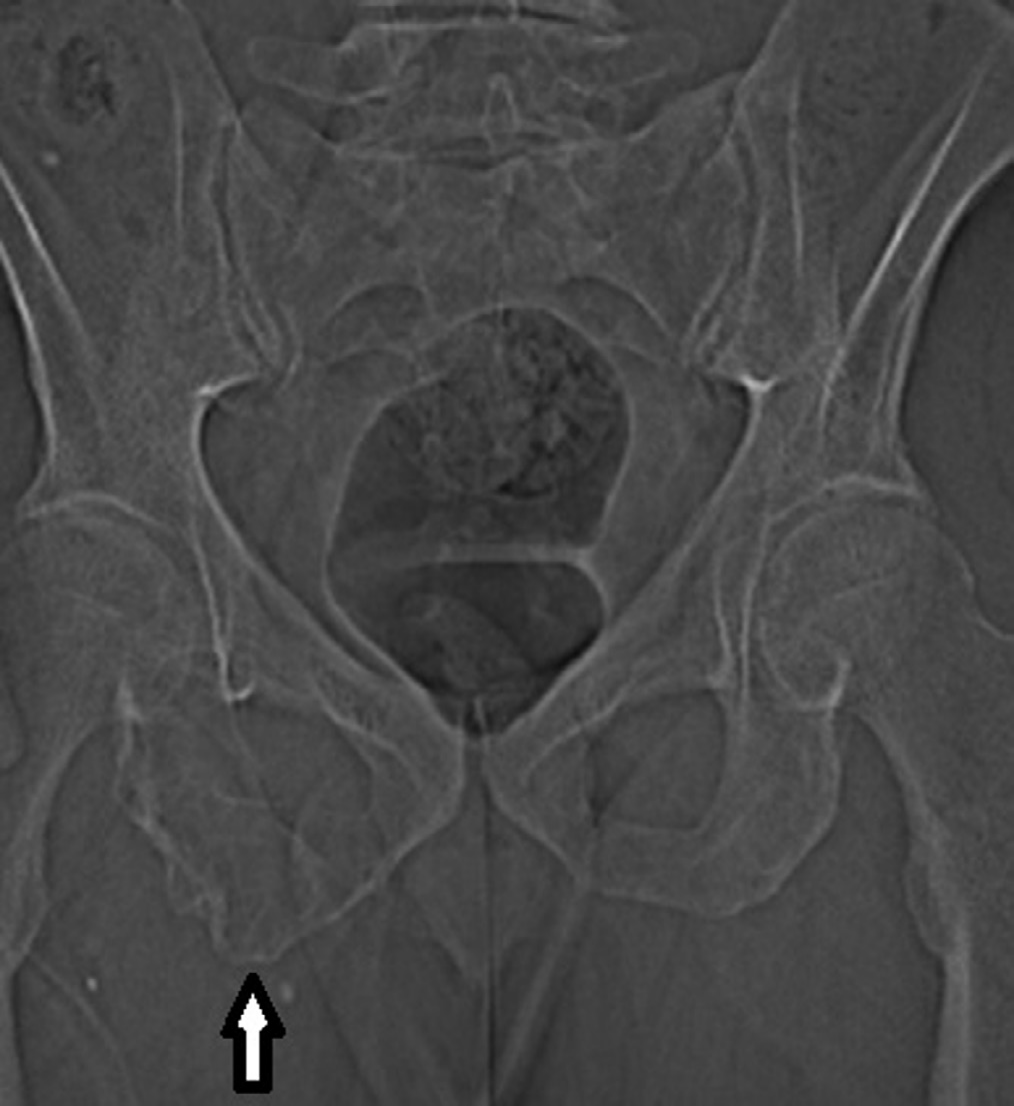
Pelvic XRays was performed (Figure 1) showing an osteolytic lesion of the right ischio pubic ramus.

Pelvic X-rays was performed (Figure 1) showing an osteolytic lesion of the right ischio pubic ramus.

**Figure 2.**
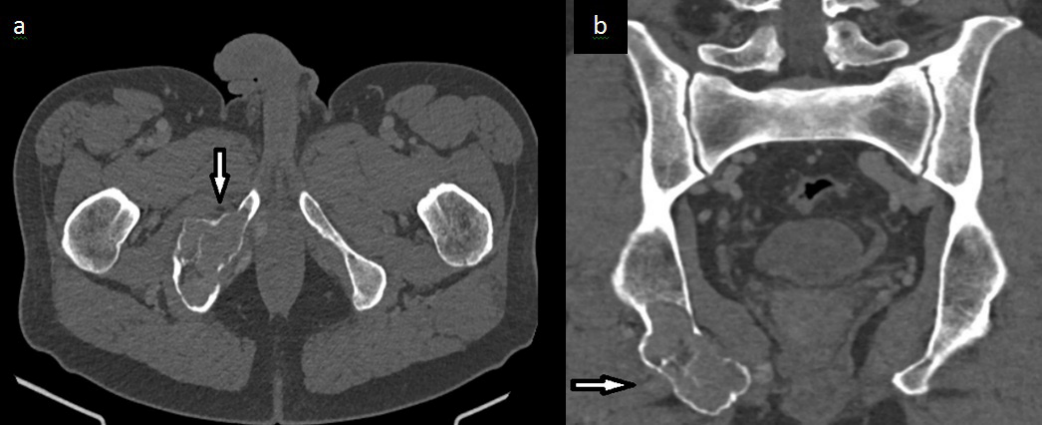
Axial and coronal pelvic CT scans in the bone window showing an osteolytic lesion of the right ischio pubic ramus (arrow) destroying the underlying cortical bone, without periosteal reaction.

CT scans (Figure 2) showed the osteolytic lesion destroying the underlying cortical bone, without periosteal reaction.

**Figure 3.**
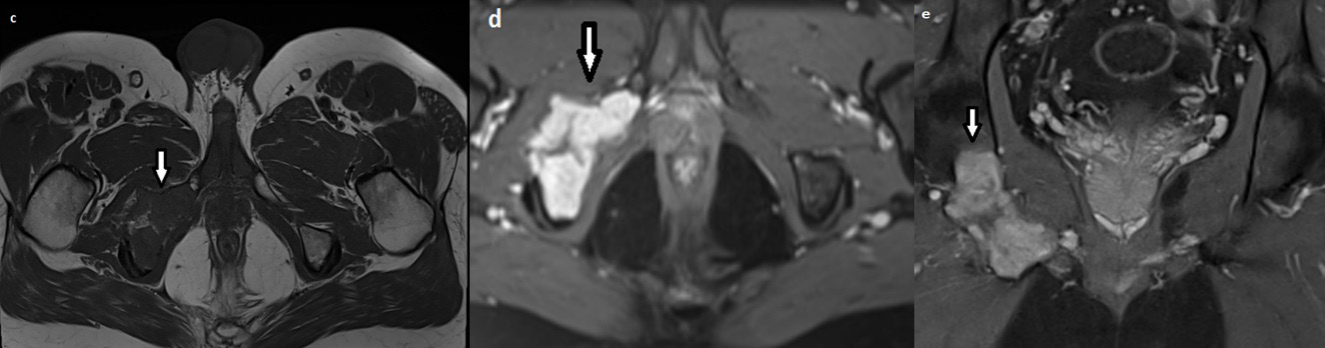
Pelvis MRI on axial T1 WS (**c**), axial (**d**) and coronal (**e**) T1 FS with gadolinium injection showing a lesion on the right ischio pubic ramus isointense on T1, enhanced after gadolinium injection with cortical destruction and soft tissue extension (arrow).

On MRI (Figure 3), the lesion was isointense on T1WS, hyperintense on diffusion WS, enhanced after gadolinium injection with cortical rupture and soft tissue invasion.

**Figure 4.**
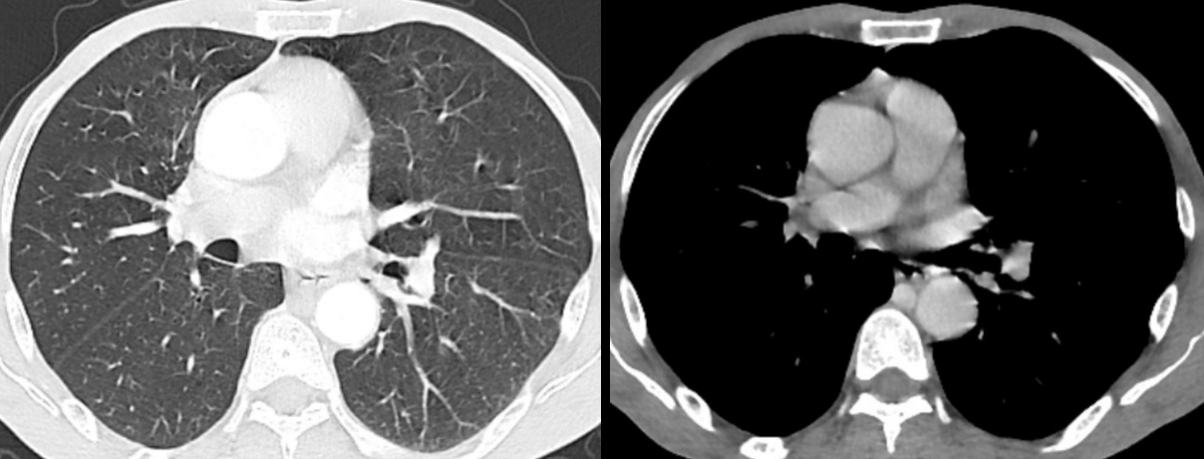
Axial CT scans of the chest in lung parenchyma and mediastinal window showing no lung lesion and no mediastinal lymphadenopathy.

The thoraco abdominal and pelvic CT scans showed no progressive lesion. There was no pulmonary parenchymal lesion or mediastinal lymphadenopathy (Figure 4).

**Figure 5.**
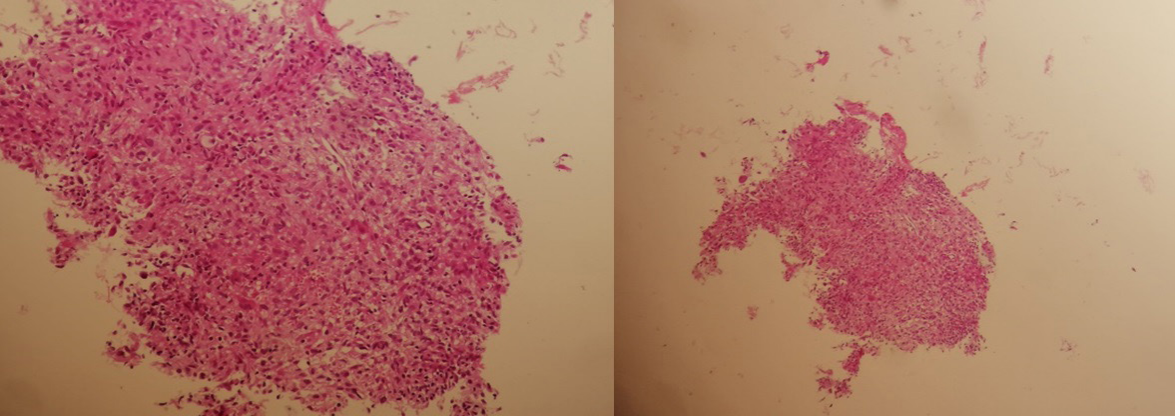
Histological analysis of the pubic and ischial lesion showing a granulomatous inflammatory infiltrate of the bone tissue made of follicles of variable size without a necrosis spot compatible with sarcoidosis.

A biopsy of the lesion was realised and histological analysis showed a granulomatous inflammatory infiltrate of the bone tissue made of follicles of variable size without a necrosis spot compatible with sarcoidosis (Figure 5).

The patient was given prednisone corticosteroid at 30 mg daily. The evolution was marked by the regression of the inflammatory syndrome and clinical symptoms.

## Discussion

Sarcoidosis (Besnier–Boeck–Schaumann’s disease) is a systemic granulomatous disease of unknown aetiology described for the first time by Jonathan Hunchinson in 1869 who reported a case of skin sarcoidosis.

Bone sarcoidosis is rare. The first description of the bone lesions was made by Jüngling in 1920, under the name of “osteitis tuberculosa multiplex cystica”.^1^

The incidence of the osseous involvement in bone sarcoidosis is ranging from 3 to 13%.^2^

The histologic lesion of sarcoidosis is a granuloma with epithelial and giant cells without caseous necrosis, which differentiates it from the tubercular granuloma. Sarcoidal granulomas infiltrate the spongy bone, destroy some bone trabeccles and thicken others. They demolish the cortical bone, giving a “moth-eaten” appearance. However, bone damage does not cause a periosteal reaction.

Bone localization is often multiple. Unique localization is rare, as is our observation.^3^

Clinically, bone lesions are often asymptomatic. They can be revealed by bone pain or discovered during routine radiological examinations.

Imaging plays an important role in the diagnosis. Bone lesions may interest the entire skeleton, but they mainly concern the short tubular bones of the hands and feet.^3,4^

When bone lesions are located at the small bones of the hands, diagnosis is often easier especially when associated with frequent skin lesions. However, the vertebral bone, long bones and pelvic lesions can mimic bone metastases. In MRI, they appear as multiple intramedullary lytic lesions, with rounded or irregular contours, iso-intense to the muscles on T1W sequences with variable signal on T2 WS, enhanced after gadolinium injection.^4,5^

Concerning the short trabecular bones of the hands and feet, lesions are mainly located in the distal and medium phalanges. They can be uni- or bilateral, asymmetrical. Several aspects have been described: cyst-like lesions of the phalanges, lace-like osteopenia or acro-osteolysis.^5,6^

As for long bones lesions, the most common radiographic aspect is the central lytic bone lesion, which is well circumscribed, without peripheral condensation and without cortical rupture. They are presented in MRI in the form of rounded nodules of the bone marrow in variable size and number, hypointense on T1 WS, hyperintense on T2 WS, enhanced after gadolinium injection. The nodule may have a fatty signal center, which is benign sign of involution. CT scan is not contributory and conventional radiography is usually normal.

These lesions are strongly suggestive of multiple myeloma due to the nodular aspect. Lesions can range in size from micronodules having a “starry sky” aspect to wide spinal cord lesions.^6^

Spinal involvement is often reported, especially in the thoracic-lumbar hinge, appearing as osteolytic lesions with a line of sclerosis affecting mainly the vertebral body and sometimes a pedicle. We can also find a condensation of vertebral body, multiple vertebral collapse or ivory vertebrae. An aspect of pseudospondylodiscitis, with a paravertebral spindle image, but disc preservation has been described.^3,6^

The pelvic bone lesions appears on CT scan in the form of pseudonodular intraosseous, well-circumscribed lytic lesions. On MRI, they have a low signal on T1W images, high signal on T2 WS and high signal on diffusion WS, which are not specific for sarcoidosis bone lesions or metastatic disease. In our case, the bone lesion is unique, lytic,with geographical contours, with cortical rupture and soft tissue extension.^4^

Facial bones lesions are lytic and usually concern nasal bones. Exceptionally, there may be interest on other facial bones (sinus, orbit, mandible). Computed tomography is very useful to pinpoint these lesions.^1,3,4^

The 3D 99Tc scintigraphy allows early and rapid detection of inflammation and bone granulomas and provide a precise localization of lesions.

When the bone lesions are multiple, the differential diagnosis includes bone metastases, tuberculosis, lymphoma, or common germ infections. In case of a single bone lesion, the differential diagnosis arises mainly with tuberculosis, bone metastasis or bone lymphoma.

The treatment of bone sarcoidosis is based on systemic corticosteroids. The indication and dosage of corticosteroids should be discussed for each patient.^1,7^

## Conclusion

Bone sarcoidosis lesions are lytic rather than condensing but no image is really specific to bone sarcoidosis. When bone lesion is associated with mediastinal or pulmonary involvement, the diagnosis is often easy. In the case of isolated bone lesion, it is difficult to differentiate it from bone tuberculosis or metastasis, so a biopsy is indicated to make the diagnosis.

This observation of bone sarcoidosis seems original to us because of the isolated bone involvement, which is rarely indicative of the disease, and especially the radiological aspect of the bone lesion that appears to be aggressive evoking a metastatic origin.

## Learning points

- Sarcoidosis is a systemic granulomatous disease of unknown aetiology.
- Bone sarcoidosis lesions are rare and not specific.
- The lesions described in the axial skeleton and long bones are very polymorphic, often lytic, multiple, nodular, rounded, of variable size and number.
- The diagnosis is difficult when there is a single bone lesion.
